# Assessment of a baloxavir marboxil treatment protocol for high pathogenicity avian influenza in Okinawa Rails, an endangered species endemic to Japan

**DOI:** 10.1371/journal.pone.0345055

**Published:** 2026-03-23

**Authors:** Mariko Miki, Yo Shimazu, Ryo Daniel Obara, Takashi Nagamine, Yumiko Nakaya, Yoshinori Ikenaka, Hiromi Osaki, Takashi Kimura, Takahiro Hiono, Norikazu Isoda, Takao Shishido, Yoshihiro Sakoda

**Affiliations:** 1 Laboratory for Drug Discovery and Development, Shionogi & Co., Ltd., Osaka, Japan; 2 Laboratory of Microbiology, Department of Disease Control, Faculty of Veterinary Medicine, Hokkaido University, Sapporo, Hokkaido, Japan; 3 Okinawa Wildlife Federation, Uruma, Okinawa, Japan; 4 Laboratory of Toxicology, Department of Environmental Veterinary Sciences, Faculty of Veterinary Medicine, Hokkaido University, Sapporo, Japan; 5 Water Research Group, School of Environmental Sciences and Development, North-West University, Potchefstroom, South Africa; 6 Translational Research Unit, Veterinary Teaching Hospital, Faculty of Veterinary Medicine, Hokkaido University, Sapporo, Japan; 7 One Health Research Center, Hokkaido University, Sapporo, Japan; 8 Laboratory of Comparative Pathology, Department of Clinical Sciences, Faculty of Veterinary Medicine, Hokkaido University, Sapporo, Hokkaido, Japan; 9 International Collaboration Unit, International Institute for Zoonosis Control, Hokkaido University, Sapporo, Hokkaido, Japan; 10 Institute for Vaccine Research and Development (HU-IVReD), Hokkaido University, Sapporo, Hokkaido, Japan; 11 Laboratory for Drug Discovery and Disease Research, Shionogi & Co., Ltd., Osaka, Japan; Teikyo University: Teikyo Daigaku, JAPAN

## Abstract

High pathogenicity avian influenza (HPAI) is a highly infectious and lethal disease of birds that causes systemic symptoms and has been spreading globally, including in Japan. The Okinawa rail (*Hypotaenidia okinawae*), a flightless bird endemic to Japan, is classified as an endangered species on the Red List. In 2004, the Ministry of the Environment of Japan began implementing a conservation breeding program for Okinawa rails, focusing on maintaining the species’ genetic diversity, captive breeding, and reintroduction to the wild. Given the potential for significant losses due to HPAI in Okinawa rails, the establishment of a treatment protocol as a preparedness measure is essential. The aim of this study was to determine an appropriate treatment method for HPAI in Okinawa rails using baloxavir marboxil (BXM), a drug shown to be effective in an avian laboratory model of HPAI virus infection. Single oral administration of BXM at 2.5 or 12.5 mg/kg did not produce plasma concentrations sufficient to achieve the expected therapeutic efficacy. Therefore, oral administration was deemed inadequate for generating the desired pharmacological effects. Consequently, subcutaneous administration of BXM to Okinawa rails at a dose of 2.5 or 7.5 mg/kg was explored as an alternative protocol, which resulted in higher systemic exposure compared with oral administration. Furthermore, plasma concentrations were maintained at therapeutically relevant levels up to 24 hours after subcutaneous administration at 7.5 mg/kg, with mild and reversible injection site irritation the main adverse effect. Based on these results, subcutaneous administration of BXM is proposed as a viable treatment protocol for HPAI in the conservation of endangered Okinawa rails.

## Introduction

High pathogenicity avian influenza (HPAI) is caused by high pathogenicity avian influenza viruses (HPAIVs), such as H5N1 and H7N9 [1,2]. HPAI primarily affects birds and has a high mortality rate [3]. HPAIV has begun to spread globally in recent years, with infections reported in wild birds and mammals in various regions [4].

The Okinawa rail (*Hypotaenidia okinawae*) is a flightless bird endemic to the northern part of Okinawa Island, Japan. The Okinawa rail has a striking pattern of black and white stripes from its chest to belly, and red beak and legs (S1 Fig). The Okinawa rail belongs to the order *Gruiformes*, which includes cranes and rails, and is characterized by a highly restricted distribution. The species, facing a critical risk of extinction, is classified as Endangered on the Red List of the International Union for Conservation of Nature and Natural Resources due to its small population. The risk of extinction is attributed to predation by invasive carnivores, traffic accidents, and habitat loss [5]. In response, the Ministry of the Environment of Japan launched a conservation and captive breeding program for Okinawa rail in 2004. The program has included efforts to develop reintroduction and captive breeding techniques to mitigate potential population declines [6]. The program has successfully preserved the genetic diversity of the captive population, achieving levels comparable to those observed in the wild, as intended. HPAIV was detected in Okinawa Prefecture, which is inhabited by Okinawa rails, during the 2022–2023 season with infections reported in other avian species [7], raising concerns over the potential impact on the Okinawa rail population. Assessments of the susceptibility of this species to HPAIV using cellular and genomic analyses revealed mutations in the Okinawa rail MDA5 gene that may play a role in viral recognition [8]. Based on these findings, researchers have suggested that Okinawa rails mount a comparatively weaker immune response to viral infection [8]. Considering that genetic diversity has been maintained through the captive breeding program for over two decades, the potential impact of HPAI could be significant. In Japan, standard countermeasures against HPAI in captive birds typically involve isolation or culling, as no vaccination or treatment strategies have been established [9,10].

Baloxavir marboxil (BXM), an antiviral drug targeting influenza in humans, selectively inhibits the activity of cap-dependent endonuclease, an influenza virus–specific enzyme present in the polymerase acidic (PA) subunit of the influenza virus RNA polymerase complex [11,12]. Cap-dependent endonuclease is essential for the initiation of viral mRNA synthesis, and inhibition of its activity leads to the suppression of influenza virus replication [13]. Administered as a prodrug, BXM is rapidly metabolized to its active form, baloxavir acid (BXA), following oral administration in mice [14], whereas it is slowly metabolized in another mammalian species, ferrets [15]. BXA reportedly exhibits antiviral efficacy against a wide variety of influenza virus strains, which is attributed to similarity in the structure of the active site of cap-dependent endonuclease in the PA subunit among different influenza viruses [16]. Both in vitro and in vivo studies have confirmed the efficacy of BXA/BXM against H5 HPAIV infection in mammals [17,18]. A single oral administration of BXM at a dose of 2.5 mg/kg or higher reportedly produces pharmacological effects in chickens infected with HPAIV [19]. Furthermore, in a study using chickens, a common avian laboratory animal, repeated oral administration of BXM for 4 weeks resulted in a no observed adverse effect level of 62.5 mg/kg/day [20], indicating the potential for safe use in birds, including Okinawa rail. The pharmacokinetic profile of BXM/BXA can vary across avian species, just as it differs between mammalian species [12]. For instance, interspecies differences in the pharmacokinetics of carboplatin have been reported in birds [21].

In Japan, as in the United States [22] and Europe [23], veterinarians are legally permitted to use human pharmaceuticals for animal treatment at their discretion in unavoidable situations [24]. Additionally, if appropriate isolation and infection control measures are implemented, captive birds with infection or suspected infection may be subjected to treatment under a predetermined plan, at the discretion of the facility manager or attending veterinarian. Given that the Okinawa rail is an endangered species and the subject of a conservation breeding program, a preparedness plan that includes pharmaceutical treatments for HPAIV in this species using an effective antiviral drug such as BXM is highly desirable. As the pharmacokinetic parameters of BXM/BXA are thought to differ among bird species, species-specific dosage regimens and administration methods should be established.

The present study was therefore conducted to obtain pharmacokinetic and safety-related information and assess a treatment protocol for HPAI using BXM as a preparedness measure for the treatment of HPAI in Okinawa rails. Ultimately, these findings may contribute to the enhancement of biodiversity through captive breeding programs and subsequent reintroduction of endangered bird species into the wild. It should be noted that its use in poultry must be strictly avoided due to public health concerns.

## Materials and methods

### Ethical Considerations

All animal experiments were conducted with the approval of the Institutional Animal and Committee of Hokkaido University (approval 23–0127, 21–0102) and performed under the guidelines of the Institutional Animal and Committee of Hokkaido University, certified by the Association for Assessment and Accreditation of Laboratory Animal Care International since 2007. In addition, the studies were conducted with the approval of the Ministry of the Environment’s committee responsible for the Okinawa rail protection and recovery program. The chickens used in the experiment were humanely euthanized by an overdose of thiopental prior to necropsy. Predefined humane endpoints were established for both species: for chickens, to determine the need for euthanasia, and for Okinawa rails, to guide rescue interventions due to their endangered status. No birds reached these endpoints during the studies. The criteria included decreased activity in Okinawa rails and a moribund condition in chickens, based on clinical signs, when recovery was deemed impossible.

### Animals

Fifteen Okinawa rails (*Hypotaenidia okinawae*), aged 2–12 years, including both males and females, were used for all experiments. Study individuals were selected from the captive population based on the following criteria: relatively stable health status, individual characteristics that allowed handling with minimal stress during experimental procedures, low reproductive priority within the captive breeding program of Okinawa rails, and voluntary acceptance of the provided diet (pellets or smelt). Individuals not included in the active breeding plan were selected. Each bird was housed individually in a cage enriched with a tree branch, fallen leaves, and a bird bath to simulate their natural habitat. The Okinawa rails used in this study had been rescued from traffic accidents in previous years or obtained through conservation breeding programs authorized by the Ministry of the Environment of Japan. Healthy birds were selected by a veterinarian caring for them daily. The birds were kept under managed care for conservation and breeding purposes at a facility owned by the Ministry of the Environment and the nonprofit organization Conservation & Animal Welfare Trust, Okinawa (Okinawa, Japan). For the supplementary local irritation study, fifteen 10-week-old chickens (*Gallus gallus domesticus*) were examined at Hokkaido University (see Supplementary Single Subcutaneous Irritation Study in Chickens). Each chicken was housed individually in a cage enriched with toys. The procedures in the following studies were carried out by research members who were familiar with the daily behavior of chickens or Okinawa rails and had extensive experience in conducting experiments with each species. Because the Okinawa rail is an endangered species under strict conservation constraints and this study aimed to broadly characterize its pharmacokinetics, only three birds per group were used as the minimum feasible sample size. Therefore, the results obtained from Okinawa rails represent a broad characterization of pharmacokinetics and should be interpreted within this context.

### Single Oral Administration Study in Okinawa Rails

#### Test Substance Preparation.

BXM tablets (20 mg Tablets, Shionogi & Co., Ltd., Osaka, Japan) were ground using a mortar and pestle.

### Study design

Nine Okinawa rails (*Hypotaenidia okinawae*) aged 2–12 years were used in the study and randomly divided into three groups (A–C). Powdered BXM was orally administered via the regular diet (namely, smelt and ibis pellets). The three groups were established based on the method of administration, as follows. In Group A, the prescribed amount of powdered BXM was directly placed into the mouth of smelts and fed to the birds. In Group B, powdered BXM was mixed with ibis pellets and fed to the birds. In Group C, powdered BXM was placed into a capsule, which was then placed into the mouth of a smelt and swallowed.

Dose-response relationships were assessed using a high and a low dose. The low dose of 2.5 mg/kg was previously reported as effective in chickens [19], and the high dose of 12.5 mg/kg was confirmed to be safe in chickens [20]. The amount of drug for administration was calculated based on body weight measured the day before administration. After oral administration of 2.5 mg/kg, a washout period of approximately 1 week was set before the same birds were administered 12.5 mg/kg.

### Examination

Clinical observations were conducted once daily for 2 days after each administration. For determination of the plasma drug concentration, blood samples (approximately 230 µL per time point) were collected from the medial metatarsal vein at 2, 8, 24, and 48 hours (h) post-administration (S2 Fig). The collected blood samples were placed in 1.5-mL tubes containing 13.8 µL of enzyme inhibitor/anticoagulant mixture (60 mmol/L dichlorvos [FUJIFILM Wako Pure Chemical Corp., Osaka, Japan]/sodium heparin [Nipro Corp., Osaka, Japan]), mixed thoroughly, and centrifuged to obtain plasma. Plasma drug concentrations were determined using liquid chromatography–tandem mass spectrometry with an internal standard method and multiple quality control levels (analyte: BXM and BXA) (S1 Method). Plasma concentrations (ng/mL) for each sample were calculated using Excel software (Microsoft Corp., Redmond WA, USA). The lower limit of quantitation (LLOQ) values were 0.8 ng/mL for both BXA and BXM. The maximum plasma concentration (C_max_, ng/mL), area under the plasma concentration–time curve (AUC) from 0 to 48 h (AUC_0-48h_, ng*h/mL), determined using the linear trapezoidal method, and time to maximum plasma concentration (T_max_, h) were calculated for each individual bird via non-compartmental analysis using PKanalix software (version 2024R1; Simulations Plus, Inc., Lancaster, CA, USA). Means and standard deviation values were calculated using Excel software. In accordance with the study protocol, below the limit of quantitation (BLQ) values were treated as zero in the analysis, as in previous BXM studies. This conservative approach is grounded in the assumption that concentrations below the quantifiable limit reflect the absence of the drug and is widely endorsed by regulatory authorities [25].

### Single Subcutaneous Administration Study in Okinawa Rails

#### Test Substance Preparation.

On the day of administration, BXM tablets (20 mg Tablets, Shionogi & Co., Ltd.) were ground using a mortar and pestle and suspended in lactic acid solution (Otsuka Pharmaceutical Factory, Tokushima, Japan) at a concentration of 1.5 or 2 mg/mL.

### Study design

Six Okinawa rails (*Hypotaenidia okinawae*) aged 4–12 years were used and randomly divided into two groups. Birds were administered a dose of 2.5 or 7.5 mg/kg, with an administration volume of 1.25 or 5.0 mL/kg, respectively. The administration volumes were calculated based on body weight measured the day before administration, and a single subcutaneous injection was made on the leg.

### Examination

Clinical observations were conducted once daily for 7 days after administration, with the injection site on the leg observed at each blood sampling point. For determination of plasma drug concentrations, blood samples (approximately 230 µL per time point) were collected from the medial metatarsal vein at 2, 8, 24, 72, 168, and 336 h post-administration. Blood sample processing, measurement, and analysis were conducted in the same manner described for oral administration. However, the AUC was calculated from time zero to 336 h (AUC_0-336h_, ng*h/mL), instead of AUC_0-48h_. The LLOQ values were 2.4 ng/mL for BXA and 1.2 ng/mL for BXM.

### Supplementary Single Subcutaneous Irritation Study in Chickens

Because BXM is intended for oral administration, concerns arose regarding potential local irritation following subcutaneous injection. To supplement the safety evaluation, a study in chickens was conducted before the subcutaneous study in Okinawa rails to pathologically assess local irritation and its recovery following a single subcutaneous administration. Pathological examinations of the injection site were conducted on Days 15 and 29 (2 and 4 weeks post-administration). Chickens were used as avian laboratory animals for this assessment. The day of administration was designated as Day 1.

### Test Substance Preparation

On the day of administration, BXM tablets (20 mg Tablets, Shionogi & Co., Ltd.) were ground using a mortar and pestle and suspended in saline (Otsuka Pharmaceutical Factory, Tokushima, Japan) at a concentration of 1.5 or 2 mg/mL.

### Study design

Fifteen 10-week-old chickens (n = 4 or 5/group on Day 15; n = 3/group on Day 29) (*Gallus gallus domesticus*, Julia/Julia-Lite strain; obtained from Iwamura Portly Ltd., Niigata, Japan) were divided into four groups. The chickens were administered BXM at 2.5 or 7.5 mg/kg, with corresponding administration volumes of 1.25 or 5.0 mL/kg. The administration volumes were calculated based on body weight measured the day before administration, and a single subcutaneous injection was conducted on the leg.

### Examination

Clinical observations were conducted once daily. At 2 and 4 weeks after administration (Days 15 and 29), chickens were euthanized via intravenous overdose with thiopental (150 mg/kg), followed by necropsy to assess gross pathological and histopathological changes at the injection site. For histopathology, tissue samples from the injection site were fixed in 10% neutral buffered formalin and embedded in paraffin. Sections of 3 µm thickness were prepared, stained with hematoxylin and eosin, and evaluated.

### Statistical analysis

Excel software (Microsoft Corp., Redmond WA, USA) was used for statistical analysis. Pharmacokinetic parameters obtained from individuals following single oral administration were statistically analyzed using one-way analysis of variance (ANOVA) as an exploratory approach to assess differences between dosing conditions with a significant level of p < 0.05. Given the small sample size (n = 3), p-values (p < 0.05) were interpreted cautiously, and effect sizes were calculated to clarify the magnitude of between-group differences using Hedges’ g, a standardized mean difference with correction for small-sample bias, together with corresponding 95% confidence intervals (CIs) [26].

## Results

### Single oral administration study in Okinawa Rails

Following single oral administration of BXM at 2.5 or 12.5 mg/kg via feeding of a smelt or ibis pellets, clinical observations indicated no BXM-related abnormalities in any of the Okinawa rails. With regard to pharmacokinetic parameters, the p values for BXA C_max_ were 0.10 at 2.5 mg/kg and 0.48 at 12.5 mg/kg, and the effect sizes (Hedges’ g) and their 95% CIs for BXA C_max_ were calculated to facilitate interpretation beyond p-values alone (Tables 1 and 2, and S2 and S3 Figs). The mean T_max_ of BXA was 2 h post-administration at 2.5 and 12.5 mg/kg under all dosing conditions. In all individuals, including those in the 2.5 mg/kg group, BXA plasma concentrations were measurable (≥0.8 ng/mL) at 2 h post-administration (T_max_), and C_max_ was derived from these quantifiable concentrations. At doses of 2.5 and 12.5 mg/kg, BXA became BLQ after 8 h and 24 h post-administration, respectively. The plasma concentrations of BXM were BLQ (<0.8 ng/mL) at all time points at a dose of 2.5 mg/kg and after 8 h post-administration at a dose of 12.5 mg/kg, showing a lower value compared with BXA in all individuals. Although one individual (animal number: C-152) exhibited lower exposure compared to other birds in the same group at 2.5 and 12.5 mg/kg, BXA concentration-time profiles showed overall similar shapes among all birds, and no markedly atypical pharmacokinetic profiles were observed (Tables 1 and S2 and S3 Figs).

**Table 1:** Baloxavir acid (BXA, active form) plasma concentration and parameters in the single oral administration study in Okinawa rails (*Hypotaenidia okinawae*).

Group	Feed containing test substance	Dose (mg/kg)	Dosing route		Time post-administration (h)				AUC_0-48h_ [Range ^a^] (ng*h/mL)	C_max_ [Range ^a^] (ng/mL)	T_max_ (h)
					2	8	24	48			
					Plasma concentration of BXA (ng/mL)						
A	Smelt	2.5	PO	Mean	1.88	BLQ ^b^	BLQ	BLQ	7.54 [5.26–9.88]	1.88 [1.31–2.47]	2.0
				SD	0.579	NC ^c^	NC	NC	2.31	0.580	0.0
				n	3	3	3	3	3	3	3
		12.5	PO	Mean	9.18	0.981	BLQ	BLQ	47.5 [44.5–49.5]	9.18 [8.78–9.43]	2.0
				SD	0.349	0.114	NC	NC	2.65	0.348	0.0
				n	3	3	3	3	3	3	3
B	Pellets	2.5	PO	Mean	1.04	BLQ	BLQ	BLQ	4.17 [3.65–4.50]	1.04 [0.912–1.12]	2.0
				SD	0.114	NC	NC	NC	0.456	0.114	0.0
				n	3	3	3	3	3	3	3
		12.5	PO	Mean	12.3	1.92	BLQ	BLQ	70.5 [47.9–87.9]	12.3 [8.83–15.2]	2.0
				SD	3.24	0.692	NC	NC	20.5	3.24	0.0
				n	3	3	3	3	3	3	3
C	Capsule in smelt	2.5	PO	Mean	2.36	BLQ	BLQ	BLQ	9.45 [5.54–12.8]	2.36 [1.38–3.21]	2.0
				SD	0.919	NC	NC	NC	3.67	0.923	0.0
				n	3	3	3	3	3	3	3
		12.5	PO	Mean	10.3	1.15	BLQ	BLQ	53.7 [22.4–73.3]	10.3 [5.61–13.6]	2.0
				SD	4.16	0.993	NC	NC	27.4	4.16	0.0
				n	3	3	3	3	3	3	3

The sampling times were 2, 8, 24, and 48 hours (h) after administration. Parameters, including maximum plasma concentration (C_max_), area under the plasma concentration–time curve from 0 to 48 h (AUC_0-48h_), and time to maximum plasma concentration (T_max_), were calculated using PKanalix software. The doses (2.5 and 12.5 mg/kg) were administered orally (per os, PO) via regular feed (smelt or pellets) to three birds per group, with dose escalation at an approximately 1-week washout interval.

a Range: Individual value ranges [in square brackets] in the mean row.

b BLQ (in this table): Below the limit of quantification (BLQ) for all individuals in the group.

c NC: Not calculated due to BLQ for all individuals.

**Table 2:** Baloxavir marboxil (BXM, prodrug) plasma concentration and parameters in the single oral administration study in Okinawa rails (*Hypotaenidia okinawae*).

Group	Feed containing test substance	Dose (mg/kg)	Dosing route		Time post-administration (h)				AUC_0-48h_ [Range ^a^] (ng*h/mL)	C_max_ [Range ^a^] (ng/mL)	T_max_ (h)
					2	8	24	48			
					Plasma concentration of BXM (ng/mL)						
A	Smelt	2.5	PO	Mean	BLQ ^b^	BLQ	BLQ	BLQ	NC ^c^	NC	NA ^d^
				SD	NC	NC	NC	NC	NC	NC	NA
				n	3	3	3	3	NC	NC	NA
		12.5	PO	Mean	1.02	BLQ	BLQ	BLQ	4.68 [0–10.4]	1.02 [0–2.13]	NA
				SD	1.07	NC	NC	NC	5.25	1.07	NA
				n	3	3	3	3	3	3	NA
B	Pellets	2.5	PO	Mean	BLQ	BLQ	BLQ	BLQ	NC	NC	NA
				SD	NC	NC	NC	NC	NC	NC	NA
				n	3	3	3	3	NC	NC	NA
		12.5	PO	Mean	0.399	BLQ	BLQ	BLQ	1.60 [0–4.79]	0.399 [0–1.20]	NA
				SD	0.691	NC	NC	NC	2.77	0.691	NA
				n	3	3	3	3	3	3	NA
C	Capsule in smelt	2.5	PO	Mean	BLQ	BLQ	BLQ	BLQ	NC	NC	NA
				SD	NC	NC	NC	NC	NC	NC	NA
				n	3	3	3	3	NC	NC	NA
		12.5	PO	Mean	BLQ	BLQ	BLQ	BLQ	NC	NC	NA
				SD	NC	NC	NC	NC	NC	NC	NA
				n	3	3	3	3	NC	NC	NA

The sampling times were 2, 8, 24, and 48 hours (h) after administration. Parameters, including maximum plasma concentration (C_max_), area under the plasma concentration–time curve from 0 to 48 h (AUC_0-48h_), and time to maximum plasma concentration (T_max_), were calculated using PKanalix software. The doses (2.5 and 12.5 mg/kg) were administered orally (PO) via regular feed (smelt or pellets) to three birds per group, with dose escalation at an approximately 1-week washout interval.

a Range: Individual value ranges [in square brackets] in the mean row.

b BLQ: Below the limit of quantification (BLQ) for all individuals in the group.

c NC: Not calculated due to BLQ for all individuals.

d NA: Not applicable due to the presence of individuals who were BLQ at all time points.

Single Subcutaneous Administration Study in Okinawa Rails

**Fig 1:**
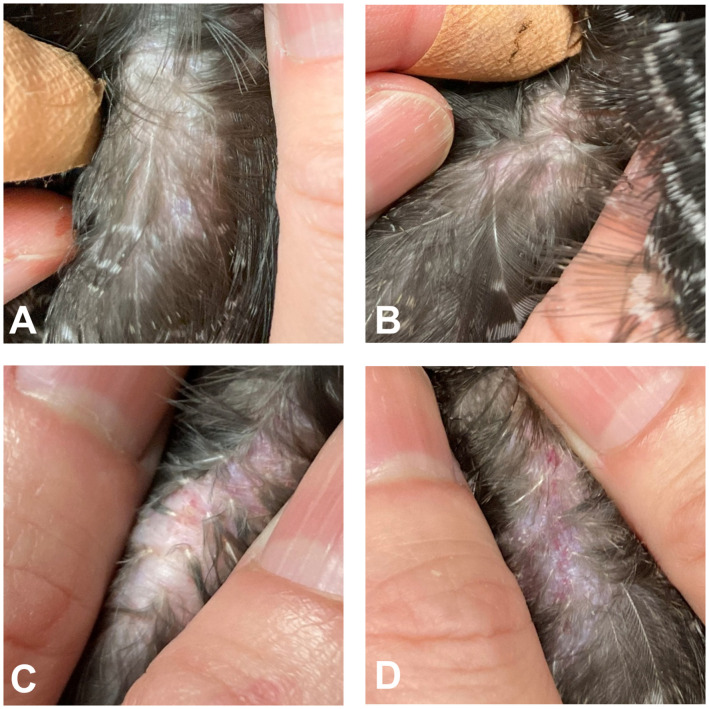
Injection site findings 24 hours after a single subcutaneous administration in Okinawa rails. The untreated leg served as a control **(A)**. Minimal redness at the injection site in one of three birds (bird number A-98) at 2.5 mg/kg **(B)**, minimal redness at the injection site in one of three birds (bird number B-125) at 7.5 mg/kg **(C)**, and mild abrasions at the injection site in one of three birds (bird number B-143) at 7.5 mg/kg (D) were observed. These findings were not observed at the subsequent sampling time (72 h after administration).

After analysis of pharmacokinetic parameters following single oral administration of BXM in Okinawa rails, an alternative route of administration capable of providing pharmacological efficacy was explored. Following single subcutaneous administration of BXM at 2.5 and 7.5 mg/kg in Okinawa rails, no BXM-related abnormalities were observed in any of the birds during clinical observation, including gait-related abnormalities. However, redness or abrasions were observed at the injection site at 24 h post-administration in birds administered BXM at either 2.5 or 7.5 mg/kg (Fig 1B–D) versus the control (untreated) leg (Fig 1A). Minimal redness at the injection site was observed in one of three rails administered BXM at 2.5 mg/kg (bird numberA-98), whereas minimal injection site redness was observed in one of three birds (bird number B-125) and mild abrasions in one of three birds (bird number B-143) administered BXM at 7.5 mg/kg, suggesting this was an administration-related change. These changes were not observed at the subsequent blood sampling point (72 h post-administration).

With regard to pharmacokinetic parameters, the mean T_max_ of BXA was 6 h and 8 h post-administration at 2.5 and 7.5 mg/kg, respectively (Tables 3 and 4, and S4 Fig). At 2.5 mg/kg, the mean C_max_ value of birds that received a single subcutaneous administration was approximately 15-fold higher than that of birds treated via oral administration (Hedges’g = 5.7; 95% CI: 2.9–8.6). The plasma concentrations of BXM were lower than that of BXA by 24 h post-administration and BLQ after 72 h post-administration.

**Table 3:** Baloxavir acid (BXA, active form) plasma concentration and parameters in the single subcutaneous administration study in Okinawa rails (*Hypotaenidia okinawae*).

Dose (mg/kg)	Dosing route		Time post-administration (h)						AUC_0-336h_ [Range ^a^] (ng*h/mL)	C_max_ [Range ^a^] (ng/mL)	T_max_ (h)
			2	8	24	72	168	336			
			Plasma concentration of BXA (ng/mL)								
2.5	SC	Mean	22.6	25.8	11.4	3.71	BLQ ^b^	BLQ	827 [617–967]	28.7 [18.9–37.9]	6.0
		SD	13.6	5.99	3.59	0.607	NC ^c^	NC	185	9.53	3.5
		n	3	3	3	3	3	3	3	3	3
7.5	SC	Mean	39.8	61.3	30.2	8.53	3.26	BLQ	2572 [2171–3092]	61.3 [47.9–82.7]	8.0
		SD	10.2	18.7	6.59	1.10	0.774	NC	472	18.7	0.0
		n	3	3	3	3	3	3	3	3	3

The sampling times were 2, 8, 24, 72, 168, and 336 hours (h) after administration. Parameters, including maximum plasma concentration (C_max_), area under the plasma concentration–time curve from 0 to 336 h (AUC_0-336h_), and time to maximum plasma concentration (T_max_), were calculated using PKanalix software. A dose of 2.5 or 7.5 mg/kg of BXM was administered subcutaneously (SC) into the leg to three birds per group.

a Range: Individual value ranges [in square brackets] in the mean row.

b BLQ: Below the limit of quantification (BLQ) for all individuals in the group.

c NC: Not calculated due to BLQ for all individuals.

**Table 4:** Baloxavir marboxil (BXM, prodrug) plasma concentration and parameters in the single subcutaneous administration study in Okinawa rails (*Hypotaenidia okinawae*).

Dose (mg/kg)	Dosing route		Time post-administration (h)						AUC_0-336h_ [Range ^a^] (ng*h/mL)	C_max_ [Range ^a^] (ng/mL)	T_max_ (h)
			2	8	24	72	168	336			
			Plasma concentration of BXM (ng/mL)								
2.5	SC	Mean	8.63	5.47	0.993	BLQ ^b^	BLQ	BLQ	94.8 [31.8–143]	8.93 [5.74–12.2]	4.0
		SD	3.26	3.07	0.889	NC ^c^	NC	NC	57.2	3.21	3.5
		n	3	3	3	3	3	3	3	3	3
7.5	SC	Mean	20.7	20.2	6.04	BLQ	BLQ	BLQ	353 [187–505]	22.2 [13.8–32.4]	4.0
		SD	7.04	11.8	2.59	NC	NC	NC	159	9.42	3.5
		n	3	3	3	3	3	3	3	3	3

The sampling times were 2, 8, 24, 72, 168, and 336 hours (h) after administration. Parameters, including maximum plasma concentration (C_max_), area under the plasma concentration–time curve from 0 to 336 h (AUC_0-336h_), and time to maximum plasma concentration (T_max_), were calculated using PKanalix software. A dose of 2.5 or 7.5 mg/kg of BXM was administered subcutaneously (SC) into the leg to three birds per group.

a Range: Individual value ranges [in square brackets] in the mean row.

b BLQ: Below the limit of quantification (BLQ) for all individuals in the group.

c NC: Not calculated due to BLQ for all individuals.

### Supplementary single subcutaneous irritation study in chickens

This supplemental study in chickens was conducted before the subcutaneous study in Okinawa rails to evaluate the irritation potential of subcutaneous administration. Pathological examinations were performed on Days 15 and 29 to assess local irritation at the administration site and its eventual resolution. Following single subcutaneous administration of BXM at 2.5 or 7.5 mg/kg via the leg, clinical observations revealed no BXM-related abnormalities in any of the chickens, including gait-related abnormalities.

With regard to gross pathology at the injection site (Table 5), no abnormalities were observed on Day 15 (2 weeks post-administration) in chickens administered BXM at 2.5 mg/kg. At 7.5 mg/kg, yellowish-white material was observed in all five birds, with a surrounding red area in three of these birds. On Day 29 (4 weeks post-administration), the findings in chickens administered 2.5 mg/kg remained the same as on Day 15, with no abnormalities. However, in two of the three birds administered BXM at 7.5 mg/kg, yellowish-white material was observed at the injection site.

**Table 5:** Pathological findings at the injection site related to baloxavir marboxil (BXM) on Days 15 and 29 in the supplementary single subcutaneous irritation study in chickens.

			Number of birds with findings in gross pathology		Number of birds with findings in histopathology ^a^															
Dose (mg/kg)	Day	Number of birds	Yellowish-white material, surrounded by red area	Yellowish-white material	Necrosis				Cellular infiltration, lymphocytes, multifocal				Granuloma with crystal-like foreign body				Fibrosis			
					－	±	+	++	－	±	+	++	－	±	+	++	－	±	+	++
2.5	15	4	0	0	4	0	0	0	4	0	0	0	3	1	0	0	3	1	0	0
	29	3	0	0	3	0	0	0	3	0	0	0	3	0	0	0	3	0	0	0
7.5	15	5	3	2	3	1	0	1	1	0	2	2	1	0	2	2	5	0	0	0
	29	3	2	2	3	0	0	0	3	0	0	0	2	0	1	0	3	0	0	0

A dose of 2.5 or 7.5 mg/kg of BXM was subcutaneously administered into the leg to 4–5 birds per group for necropsy on Day 15 and 3 birds per group for necropsy on Day 29.

a  − : Normal, ± : Minimal, + : Mild, ++: Moderate

Histopathological examination of the injection site (Table 5) revealed minimal fibrosis or minimal granuloma formation with crystal-like foreign bodies on Day 15 in one of four chickens administered BXM at 2.5 mg/kg, whereas no abnormalities were observed in any of the birds on Day 29. The following findings were observed at the injection site on Day 15 in chickens administered BXM at 7.5 mg/kg: mild to moderate cellular infiltration (lymphocytes, multifocal) in four of five birds, mild to moderate granuloma formation with crystal-like foreign bodies in four of five birds, and minimal to moderate necrosis in two of five birds. On Day 29, mild granuloma formation with crystal-like foreign bodies was observed in one of three birds administered BXM at 7.5 mg/kg.

## Discussion

Given the lack of an effective treatment for HPAI in Okinawa rails, an endangered species endemic to Japan, the potential application of BXM as a therapeutic option is worth investigating. The present study was conducted to determine the pharmacokinetic profile and safety of BXM administration in Okinawa rails. Given the small sample size used in studies on endangered species, the present results have limitations for statistics and should be interpreted as providing a broad characterization of pharmacokinetics in Okinawa rails. Previous studies have suggested that BXM exhibits pharmacological effects in birds if the plasma concentration of the active form, BXA, is maintained at ≥28 ng/mL [19,20]. Based on this finding, a target plasma concentration of 28 ng/mL for BXA was considered the threshold for pharmacological effects in Okinawa rails, and the period during which the plasma concentration remained above this threshold was defined as the period of pharmacological action. The pharmacological effects of BXM in Okinawa rails were then investigated in the present study based on this criterion. As this criterion is based on data from chickens, a species known to be highly susceptible to HPAIV with a high mortality rate [3], a limitation of our study is the potential for interspecies differences in pharmacodynamics [27]. Although itraconazole, an antifungal drug effective in other avian species, has been reported to have limited efficacy in Okinawa rails [28], pharmacodynamics information on BXM remains largely unavailable for this endangered species, as with other drugs. Therefore, this value (≥28 ng/mL), obtained from a highly susceptible species, should be interpreted as a tentative value, and pharmacodynamic data in avian species remain under investigation [29].

In the present analysis, BLQ values were treated as zero, a conservative approach commonly adopted in regulatory bioanalytical studies. Consistent with the analytical results, chromatographic data for BLQ samples showed no clearly quantifiable compound peaks, indicating concentrations below the LLOQ [30]. Although alternative approaches for handling BLQ data, such as omitting BLQ values or inputting them as half of the lower limit of quantification (LLOQ/2), have been existing for non-compartmental analysis, these methods may lead to overestimation of exposure and potentially misleading interpretations of pharmacological effects, particularly at lower dose levels [31]. Moreover, replacement with LLOQ/2 has been reported to be inappropriate when the proportion of BLQ data exceeds 5% [32], as observed in the present study. Consequently, while treating BLQ values as zero may result in a downward bias in AUC and C_max_, this analysis was adopted in accordance with the study protocol and previous studies to ensure a conservative and acceptable by regulatory authorities [25]. Furthermore, since all LLOQ values (oral study: 0.8 ng/mL for both BXA and BXM; subcutaneous study: 2.4 ng/mL for BXA and 1.2 ng/mL for BXM) were below the target concentration (28 ng/mL), handling of BLQ values does not influence the estimated duration above the efficacy concentration in either the oral or subcutaneous study.

The dosing conditions for oral administration thorough the regular diet were selected as a clinically feasible and normal route based on advice from veterinarians specializing in the breeding and reintroduction of Okinawa rails, while forced oral administration was avoided. During administration via the diet, birds readily consumed the entire provided diet without hesitation, and brief post-dose observations showed no evidence of regurgitation or abnormal behavior. Although continuous monitoring was not performed under naturalistic housing conditions (e.g., leaf litter and opportunities to hide), regurgitation or loss of the administered dose is unlikely to explain the low oral bioavailability. The observed inter-individual differences were considered to be within the expected biological range because the pharmacokinetic profiles were overall similar in shape, despite some inter-individual variability, including lower exposure in animal number C-152. Furthermore, although the age range of the birds included in this study was relatively broad (2–12 years), no consistent age-related trends in pharmacokinetic parameters were observed. No clear trend in pharmacokinetic results associated with dietary conditions was observed, as indicated by the effect sizes (Hedges’ g) and their 95% CIs, with one-way ANOVA showing no statistically significant differences among groups (p > 0.05). Following single oral administration of BXM at 2.5 mg/kg or 12.5 mg/kg in Okinawa rails, the mean C_max_ of BXA did not exceed 28 ng/mL under any of the dosing conditions. Consequently, it was not possible to determine an appropriate dosage regimen for Okinawa rails. Low plasma concentrations of both the active form (BXA) and prodrug (BXM) suggested poor oral absorption under this clinically feasible dosing condition, hindering the establishment of an effective oral administration protocol for Okinawa rails. Therefore, an alternative route of administration that could provide pharmacological efficacy was explored.

Oral administration is generally more susceptible to first-pass metabolism than other administration routes [33]. In ferrets, the clearance of orally administered BXM is more rapid compared with other mammals [34]. Furthermore, in the study by Lee et al. (2020) [34], plasma concentrations of BXA were higher after a single subcutaneous administration of BXA at 4 mg/kg than after oral administration of BXM at 10 mg/kg. Subcutaneous administration is known to result in slower absorption and prolonged exposure compared with other routes [33]. In a study in mice, BXA exhibited more sustained plasma exposure following a single subcutaneous administration than after a single oral administration of BXM [14]. These findings suggested that subcutaneous administration, which is commonly utilized in avian species, could be suitable for treating HPAI in Okinawa rails using BXM.

After subcutaneous administration of BXM at 7.5 mg/kg, plasma concentrations exceeded the target level for up to 24 h. Furthermore, the mean C_max_ of BXA following subcutaneous administration at 2.5 mg/kg was higher than that observed following oral administration at 12.5 mg/kg. These results suggest that subcutaneous administration is more likely to be effective than oral administration in this species. In addition, these data suggest that a single daily subcutaneous administration is suitable to provide pharmacological efficacy in Okinawa rails. The concentration of the prodrug, BXM, was lower than that of BXA, indicating that the prodrug is converted to the active form following subcutaneous administration.

Although subcutaneous administration of BXM in Okinawa rails was expected to provide pharmacological efficacy, minimal local changes suggesting a mild irritation were observed at the injection site at 24 h post-administration. These reactions were considered to be either administration-related changes or self-inflicted injuries due to discomfort at the injection site [35]. However, none of these changes were observed at 72 h post-administration, the subsequent blood sampling point. Despite mild irritation at the injection site, no abnormalities, including gait-related abnormalities, were observed, indicating that the injection-site changes did not pose a threat to survival.

As BXM is intended for oral administration, local irritation following subcutaneous injection was of concern. The potential for development and resolution of local irritation was therefore conducted in chickens. Pathological examinations were conducted on Days 15 and 29. In the 2.5 mg/kg group, minimal fibrosis and granulomatous changes suggestive of a mild foreign body reaction were observed on Day 15. Compared with Day 15, the histological changes on Day 29 in chickens administered BXM at 2.5 mg/kg were suggestive of recovery. In the 7.5 mg/kg group, a yellowish-white material, presumed to be the administered substance, was observed on gross evaluation on Day 15, and this material was accompanied by a surrounding red area. Histopathological examination on Day 15 revealed the development of granulomas as a biological response to the foreign material, along with changes indicative of irritation, including necrosis and associated inflammation. Compared with Day 15, gross and histopathological findings on Day 29 were suggestive of recovery in chickens administered BXM at 7.5 mg/kg. Thus, although a biological response to the injected foreign material and dose-dependent irritative changes were observed on Day 15, these changes were reversible. Although histopathological changes were observed, no impairment in clinical observation, including posture, gait, or food intake, was detected throughout the observation period until necropsy. In toxicological evaluations, mild, adaptive, and transient responses without any functional impairment observed in the present study would be generally considered non-adverse under the condition of the present study, even when they are test substance-related changes [36]. Since repeated subcutaneous administration at the same site was not conducted, the effects of repeated dosing remain unknown and warrant cautious interpretation.

The results in this study indicate that subcutaneous administration of BXM at 7.5 mg/kg can provide pharmacological efficacy in Okinawa rails for up to 24 h post-administration. However, mild and reversible irritative changes at the injection site remain a concern and should be carefully monitored during BXM use, especially when repeatedly used. When administration is repeated for up to approximately 7 days, corresponding to the reported shedding duration of HPAIV [37], rotation of injection sites (e.g., right or left leg, medial or lateral aspects) is recommended to minimize local tissue damage and to avoid interference with recovery at the injection site. It is also recommended to confirm that no signs suggestive of local irritation remain, or that any such changes at previously used injection sites have been resolved before subsequent administrations. In addition, veterinarians providing treatment should be aware that rescue procedures such as repeated capture or injections may cause stress in wildlife or endangered species; therefore, BXM treatment should be given only under the supervision of specialist veterinarians and must not be used indiscriminately. In the event of HPAI infection in Okinawa rails, treatment with BXM under veterinary supervision may contribute to the conservation of this endangered and endemic species. Decisions to initiate treatment should be guided by predefined triage and treatment initiation criteria established in advance in consultation with the Ministry of the Environment, which is the competent authority for endangered species. Such decisions should consider whether the individual can tolerate the cumulative stress associated with repeated capture and injection, and should include consideration of cases in which palliative care or non-intervention may be more appropriate. Additionally, based on the histopathological findings in chickens, release decisions for wild birds should rely on comprehensive clinical evaluation of gait, mobility, feeding behavior, and body weight to minimize potential predation risk. The results of this study are expected to aid in the development of HPAI countermeasures for endangered avian species managed by the Ministry of the Environment of Japan and other organizations, and the results should also aid subsequent programs aimed at conservation and breeding and reintroduction to the wild. These results also could aid in the captive breeding and reintroduction of other endangered avian species, ultimately contributing more broadly to biodiversity conservation.

Importantly, the use of BXM should be strictly limited to therapeutic or post-exposure prophylactic purposes in endangered avian species maintained under managed care. Its use for prophylactic purposes in any unmanaged bird species during the treatment period is strongly discouraged under all circumstances. Moreover, use in poultry must be strictly avoided due to public health concerns.

## Supporting information

S1 Fig Appearance and blood sampling of Okinawa rails. Okinawa rails are characterized by striking black-and-white stripes on the chest and belly, and a red beak and legs (A–B). Blood samples in this study were obtained via the medial metatarsal vein (C).
(TIF)

S1 Method Method for liquid chromatography–tandem mass spectrometry measurement of plasma concentrations. BXM and BXA were extracted from plasma via deproteinization and separated by liquid chromatography using an L-column3 C18 column (Chemicals Evaluation and Research Institute, Japan). The column effluent was analyzed using a 6495B mass spectrometer (Agilent) at the One Health Research Center, Hokkaido University, following procedures referenced from previously reported analytical methods [38].
(PDF)

S2 Fig Plasma concentrations of baloxavir acid (BXA, active form) and baloxavir marboxil (BXM, prodrug) in the single oral administration study in Okinawa rails: mean values (A for BXA, B for BXM) and individual values (C for BXA, D for BXM). The sampling times were 2, 8, 24, and 48 hours (h) after administration. Mean data represent the mean ± SD.
(TIF)

S3 Fig Maximum plasma concentration (C_max_; plasma concentration at 2 hours post-administration) of baloxavir acid (BXA, active form) in the single oral administration study in Okinawa rails. Data represent the mean ± 95% confidence interval (CI) for each group (left panel) and the effect size (Hedges’ g) ± 95% CI for between-group comparisons (right panel).
(TIF)

S4 Fig Plasma concentrations of baloxavir acid (BXA, active form) and baloxavir marboxil (BXM, prodrug) in the single subcutaneous administration study in Okinawa rails: mean values (A for BXA, B for BXM) and individual values (C for BXA, D for BXM). The sampling times were 2, 8, 24, 72, 168, and 336 hours (h) after administration. Mean data represent the mean ± SD.
(TIF)
