# Supplementary figures and images for: Assessment of a baloxavir marboxil treatment protocol for high pathogenicity avian influenza in Okinawa Rails, an endangered species endemic to Japan
Source: PLoS One. 2026 Mar 23;21(3):e0345055. doi: 10.1371/journal.pone.0345055 (PMC13008105; doi:10.1371/journal.pone.0345055)

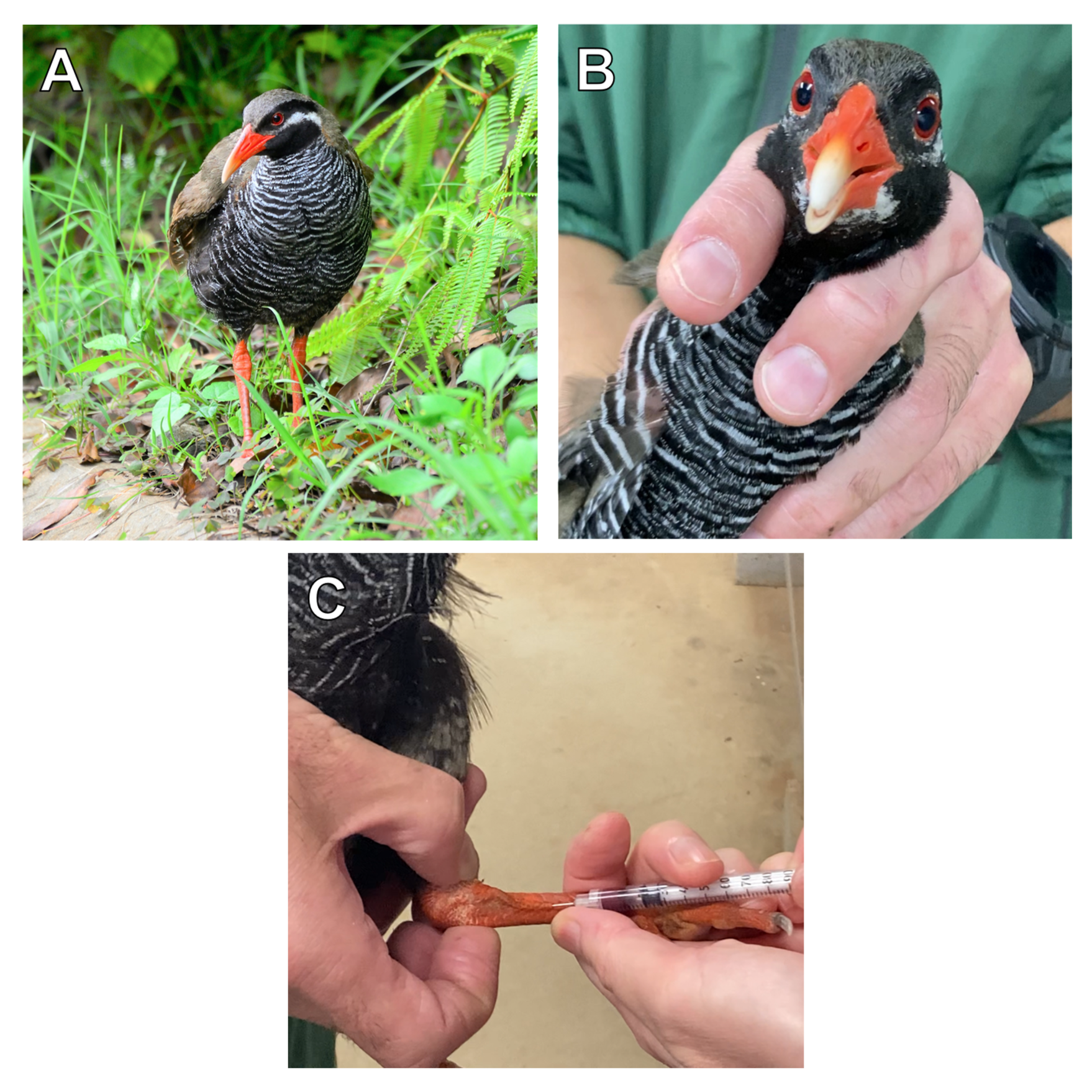

Supplement: S1 Fig — Okinawa rails are characterized by striking black-and-white stripes on the chest and belly, and a red beak and legs (A–B). Blood samples in this study were obtained via the medial metatarsal vein (C). (TIF) [file pone.0345055.s001.tif]

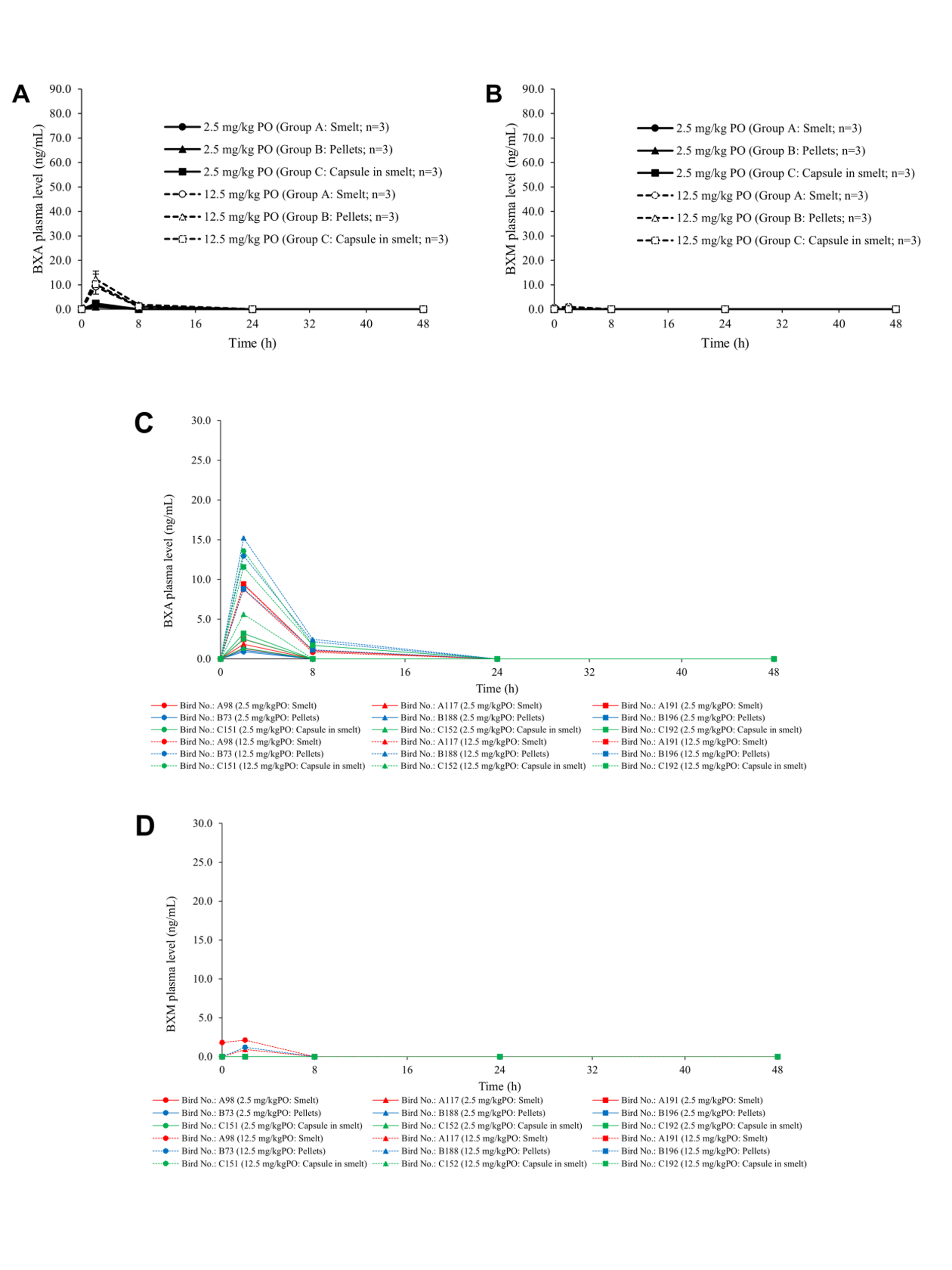

Supplement: S2 Fig — The sampling times were 2, 8, 24, and 48 hours (h) after administration. Mean data represent the mean ± SD. (TIF) [file pone.0345055.s003.tif]

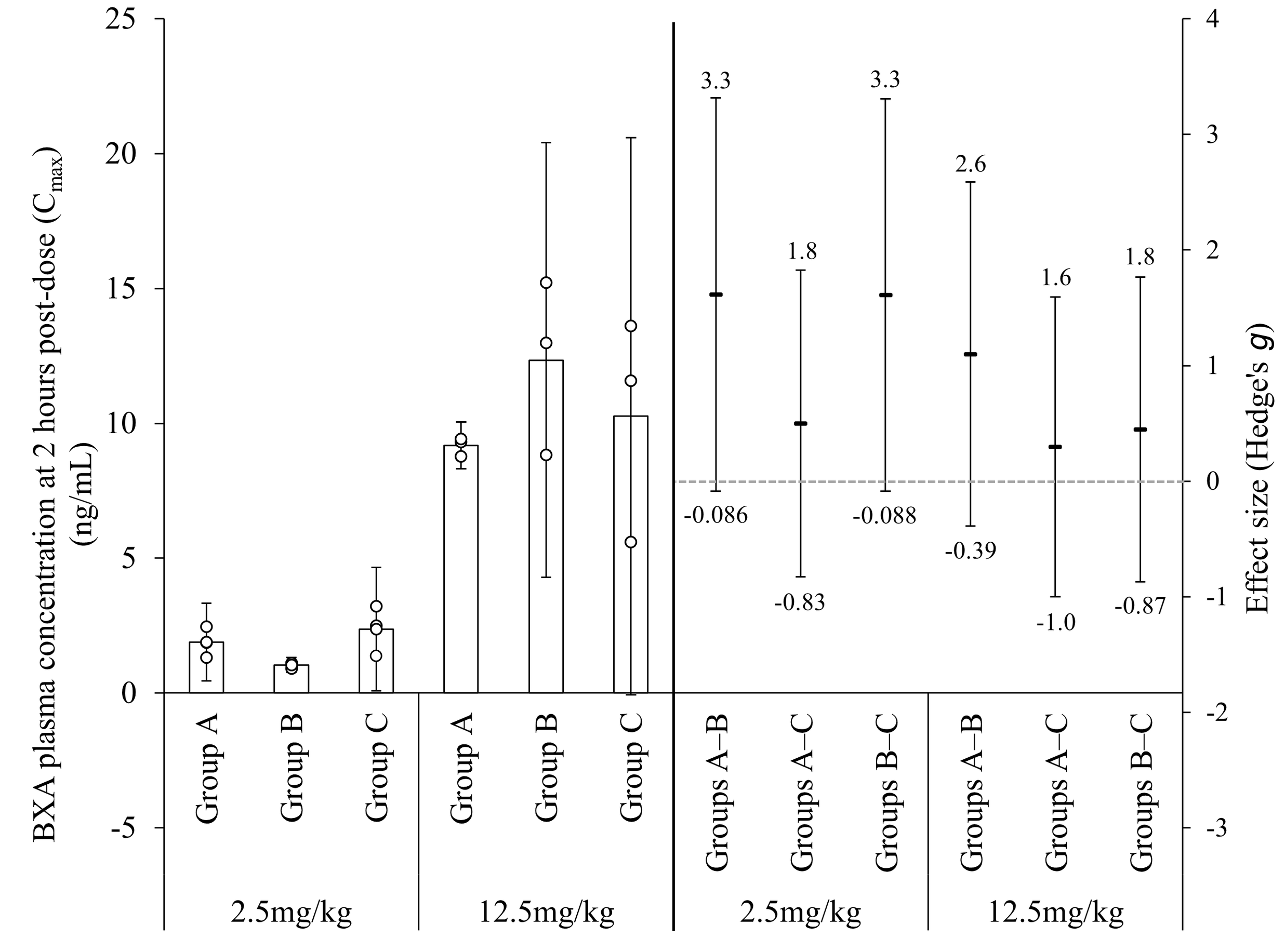

Supplement: S3 Fig — Data represent the mean ± 95% confidence interval (CI) for each group (left panel) and the effect size (Hedges’ g) ± 95% CI for between-group comparisons (right panel). (TIF) [file pone.0345055.s004.tif]

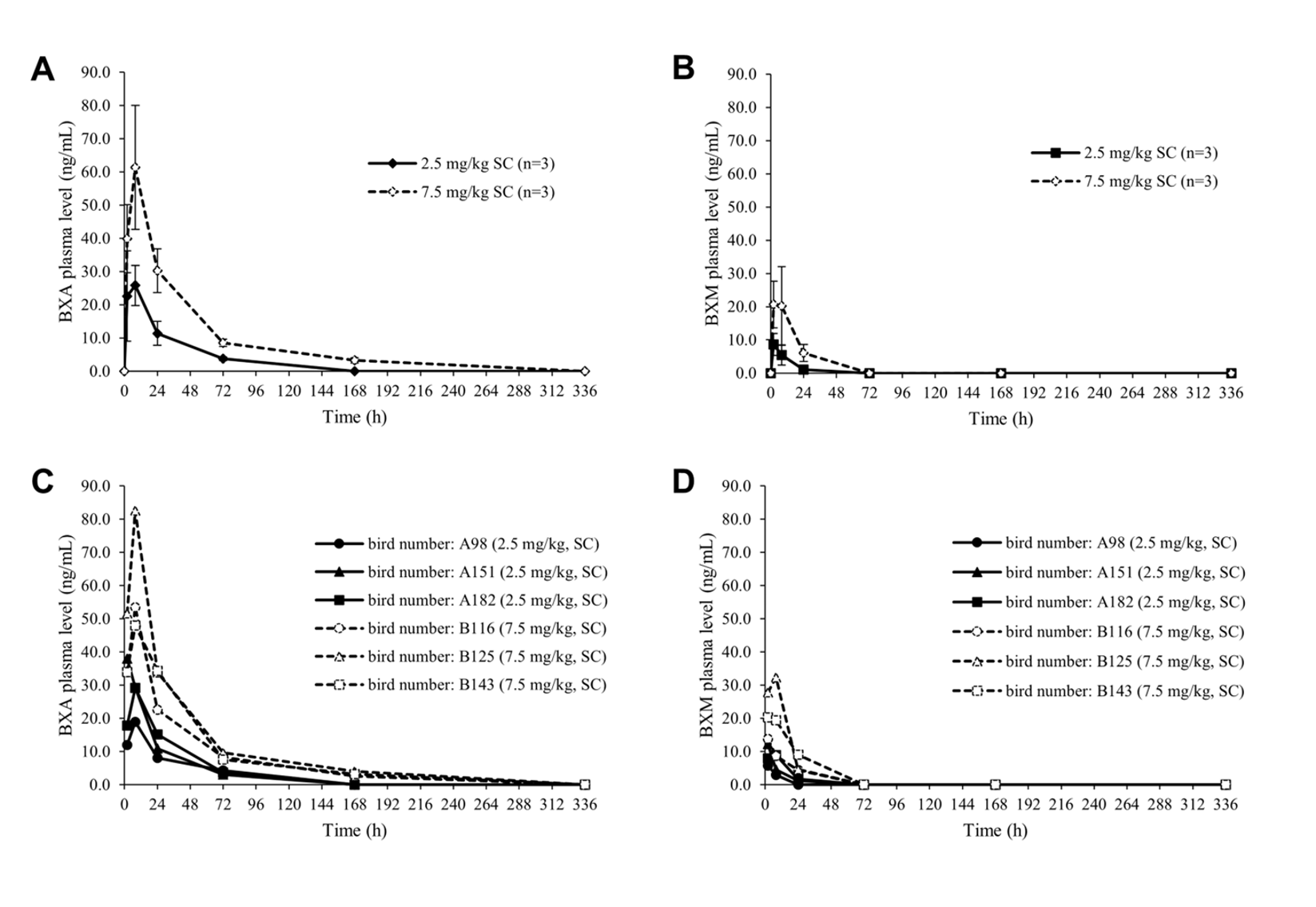

Supplement: S4 Fig — The sampling times were 2, 8, 24, 72, 168, and 336 hours (h) after administration. Mean data represent the mean ± SD. (TIF) [file pone.0345055.s005.tif]
