# Supplementary material for: Assessment of a baloxavir marboxil treatment protocol for high pathogenicity avian influenza in Okinawa Rails, an endangered species endemic to Japan
Source: PLoS One. 2026 Mar 23;21(3):e0345055. doi: 10.1371/journal.pone.0345055 (PMC13008105; doi:10.1371/journal.pone.0345055)
Supplement: S1 Method — BXM and BXA were extracted from plasma via deproteinization and separated by liquid chromatography using an L-column3 C18 column (Chemicals Evaluation and Research Institute, Japan). The column effluent was analyzed using a 6495B mass spectrometer (Agilent) at the One Health Research Center, Hokkaido University, following procedures referenced from previously reported analytical methods [38]. (PDF) [file pone.0345055.s002.pdf]

S1 Method: Method for liquid chromatography–tandem mass spectrometry measurement of plasma concentrations.

BXM and BXA were extracted from plasma via deproteinization and separated by liquid chromatography using an L-column3 C18 column (Chemicals Evaluation and Research Institute, Japan). The column effluent was analyzed using a 6495B mass spectrometer (Agilent) at the One Health Research Center, Hokkaido University, following procedures referenced from previously reported analytical methods [38].

[38]: Hirai A, Yamazaki R, Kobayashi A, Kimura T, Nomiyama K, Shimma S, et al. Detection of changes in monoamine neurotransmitters by the neonicotinoid pesticide imidacloprid using mass spectrometry. Toxics. 2022;10(11):696.
